# Molecular Mechanisms of Selenium Mitigating Lead Toxicity in Chickens via Mitochondrial Pathway: Selenoproteins, Oxidative Stress, HSPs, and Apoptosis

**DOI:** 10.3390/toxics11090734

**Published:** 2023-08-25

**Authors:** Weichen Hong, Yuhao Liu, Jiatian Liang, Chunyu Jiang, Meijin Yu, Wei Sun, Bin Huang, Na Dong, Lu Kang, You Tang

**Affiliations:** 1 College of Animal Science and Technology, Northeast Agricultural University, Harbin 150030, China; 2 Electrical and Information Engineering College, Jilin Agricultural Science and Technology University, Jilin City 132101, China; 3 Institute of Agricultural Quality Standards and Testing Technology, Xinjiang Academy of Agricultural Sciences, Urumqi 830091, China

**Keywords:** lead, selenium, mitochondrial pathway, Txnrds, oxidative stress, HSPs, apoptosis

## Abstract

Lead (Pb), a hazardous heavy metal, can damage the health of organisms. However, it is not clear whether Pb can damage chicken cerebellums and thalami. Selenium (Se), an essential nutrient for organisms, has a palliative effect on Pb poisoning in chickens. In our experiment, a model of chickens treated with Pb and Se alone and in combination was established to investigate the molecular mechanism of Se alleviating Pb-caused damage in both chicken cerebellums and thalami. Our morphological results indicated that Pb caused apoptotic lesions, such as mitochondrial and nuclear damage. Further, the anti-apoptotic gene Bcl-2 decreased; on the contrary, four pro-apoptotic genes (p53, Bax, Cyt c, and Caspase-3) increased under Pb treatment, meaning that Pb caused apoptosis via the p53-Cyt c-Caspase-3 pathway. Furthermore, we further demonstrated that Pb elevated four HSPs (HSP27, HSP40, HSP70, and HSP90), as well as HSP70 took part in the molecular mechanism of Pb-caused apoptosis. In addition, we found that Pb exposure led to oxidative stress via up-regulating the oxidant H_2_O_2_ and down-regulating four antioxidants (CAT, SOD, GST, and GPx). Moreover, Pb decreased three Se-containing factors (Txnrd1, Txnrd2, and Txnrd3), further confirming that Pb caused oxidative stress. Interestingly, Se supplementation reversed the above changes caused by Pb and alleviated Pb-induced oxidative stress and apoptosis. A time dependency was demonstrated for Bcl-2, Bax, and Cyt c in the cerebellums, as well as CAT, GPx, and p53 in the thalami of Pb-exposed chickens. HSP70 in cerebellums and HSP27 in thalami were more sensitive than those in thalami and cerebellums, respectively, under Pb exposure. Pb-induced apoptosis of thalami was more severe than cerebellums. In conclusion, after Pb treatment, Txnrds mediated oxidative stress, oxidative stress up-regulated HSPs, and finally, HSP70 triggered apoptosis. Se supplementation antagonized Pb-induced oxidative stress and apoptosis via the mitochondrial pathway and selenoproteins in chicken cerebellums and thalami. This study provides new information for the mechanism of environmental pollutant poisoning and the detoxification of Se on abiotic stress.

## 1. Introduction

Lead (Pb) is a heavy metal that has long been applied by human beings in industry, such as paint, batteries, metal electroplating, metal smelting, and pigments. However, Pb is toxic. The application of Pb leads to excessive Pb entering the environment, causing environmental pollution, and endangering the health of living organisms including humans and birds. A Japanese construction worker who was exposed to Pb was diagnosed as having Pb poisoning [1]. In India, children living near a battery manufacturing plant in Patna had elevated blood Pb levels, and some children showed neuropathic symptoms [2]. Descalzo et al. (2021) collected blood samples of hospitalized sick and dead birds in the wild of Spain and found that the Pb level of blood in three species (Eurasian griffon, Spanish eagles, and red kites) of birds was above 20 μg/dL, which exceeds the minimum threshold for subclinical poisoning (20–50 μg/dL) [3]. Chickens are the most-cultivated and -eaten bird. Yazdanparast et al. (2022) found that chickens raised in urban gardens in Sydney, Australia, had a high blood Pb level [4]. The cerebellum and thalamus are important parts of the nervous system. The cerebellum is a motor regulator and can maintain body balance. The thalamus is an advanced center of sensation. Two recent reports demonstrated Pb-injured rat cerebellums and thalami [5,6]. However, whether Pb can damage chicken cerebellums and thalami remains unclear. Selenium (Se), an essential nutrient element for animals, also has high health care and medicinal value and is of wide concern by human beings. Se alleviated Pb-induced chicken Leydig cell damage [7] and cerebrum apoptotic damage [8] in chickens. Thus, we hypothesized that Pb exposure can result in apoptotic injury in chicken cerebellums and thalami, and dietary Se supplementation can resist the damage.

Zhu et al. (2017) found that Pb exposure led to apoptotic damage via down-regulating B-cell lymphoma-2 (Bcl-2), up-regulating p53, Bcl-2-associated X protein (Bax), cytochrome c (Cyt c), and Caspase-3 in chicken cerebrums [8]. Nevertheless, it is unclear whether Pb can induce apoptosis in chicken cerebellums and thalami. Therefore, we assumed that Pb can cause apoptosis and wanted to observe the morphological structure and determine the anti-apoptotic gene Bcl-2 and four pro-apoptotic genes (p53, Bax, Cyt c, and Caspase-3) in chicken cerebellums and thalami. Two recent studies demonstrated that Se had a protective effect against excessive heavy-metal-induced apoptosis. Se supplementation relieved mercury-chloride-caused Bcl-2 down-regulation and apoptosis in chicken spleens [9]. Se antagonized decreased Bcl-2, increased p53, Bax, Cyt c, and Caspase-3, and apoptosis in the kidneys of chromium-exposed chickens [10]. Accordingly, we wanted to further prove that Se can alleviate Pb-induced apoptotic damage through observing the morphological structure and measuring the above apoptosis-related genes.

Researchers have found that oxidative stress occurred along with cerebellar and thalamic damage in rats. Barium exposure up-regulated Hydrogen Peroxide (H_2_O_2_), down-regulated Catalase (CAT), SOD, and Glutathione Peroxidase (GPx), and resulted in oxidative stress and cerebellar damage in rats [11]. It was reported that SOD activity decreased and oxidative stress and cerebellar damage occurred in Pb-poisoned rats [12]. Rats with schizophrenia developed oxidative stress and tissue injury in thalami [13]. However, oxidative stress is involved in the molecular mechanism of excessively Pb-damaged chicken cerebellums, and the case for animal thalami is still unknown. H_2_O_2_ is one of the members of the reactive oxygen species. CAT, SOD, GPx, and GST are antioxidant enzymes. Hence, we postulated that Pb can elevate H_2_O_2_, decrease CAT, SOD, GPx, and GST, and induce oxidative stress in chicken cerebellums and thalami. Se has the function of antioxidation. Se supplementation relieved Pb-induced oxidative stress in chicken testes [14]. Thioredoxin reductases (Txnrds) are selenoproteins, also a kind of important oxidoreductase [15], and play an antioxidant role [16]. Thus, we wanted to investigate whether Pb can induce oxidative stress from the antioxidation and oxidation point of view and whether Se can alleviate Pb-caused oxidative stress.

Shang et al. (2023) reported that HSP70 and HSP90 increased in *Luciobarbus capito* spleens under salt stress [17]. A study found that HSP27, HSP40, HSP70, and HSP90 were elevated in excessive manganese-exposed chicken embryo neural cells [18]. Tan and collaborators mentioned that Se reversed Cd-up-regulated HSP40, HSP70, and HSP90 in chicken peripheral blood neutrophils [19]. Se also inhibited Pb-increased HSP27, HSP40, HSP70, and HSP90 in chicken germ cells [7]. However, there are no reports about Pb-induced changes of HSPs in animal cerebellums and thalami. Accordingly, we also wanted to further investigate whether HSPs participated in the molecular mechanism of Se mitigating Pb poisoning in chicken cerebellums and thalami.

Taken together, we wanted to establish a model of chickens treated with Pb and Se alone and in combination and aimed to explore the complex mechanism of the alleviating effect of Se on Pb-induced apoptotic injury through observing the microstructures and ultrastructures, as well as detecting Txnrds, oxidative stress indexes, HSPs, and apoptosis-related genes in chicken cerebellums and thalami.

## 2. Materials and Methods

### 2.1. Animal Model

All procedures in the experiment were approved by the Northeast Agricultural University Institutional Animal Care and Use Committee with Approval Number SRM-06. The Hyline chicks (healthy males, one-day-old) used in our experiment came from Weiwei company (Harbin, China), were kept in a strictly sterilized environment at the Experimental Animal Center, College of Animal Medicine, Northeast Agricultural University (Harbin, China), and were fed drinking water and a standard commercial diet containing 0.49 mg/kg Se. After an adaption period of 7 days, 180 chicks were randomly allocated into four groups (the control group, the Se group, the Pb group, and the Se/Pb group). Each group consisted of three replicates with fifteen chicks per replicate. Pb was added to the drinking water of the Pb and Se/Pb groups in the form of (CH_3_COO)_2_Pb·3H_2_O. The Pb concentration in the drinking water was 350.0 mg/L based on median lethal does (LD_50_) of Pb to chickens [20]. Se was added to the diet of the Se and Se/Pb groups in the form of Na_2_SeO_3_·5H_2_O, and the Se content in the diet was 1.0 mg/kg. (CH_3_COO)_2_Pb·3H_2_O (analytical-reagent-grade, 99.5%) and Na_2_SeO_3_·5H_2_O (analytical-reagent-grade, 98%) were purchased from Tianjinzhiyuan Chemical Reagent Co., Ltd. (Tianjin, China). All chickens were free to feed and drink water.

### 2.2. Sample Collection

On Days 30, 60, and 90, fifteen chickens in each group were randomly selected and euthanized. The cerebellums and thalami were quickly removed and rinsed with cold sterile deionized water. The obtained samples were treated as follows. Some samples were homogenized for the detection of oxidative stress indexes. Some samples were cut into small 1 cm^3^ pieces and fixed immediately with 4% (*v*/*v*) paraformaldehyde at room temperature for microstructural observation. Some samples were fixed immediately with 2.5% (*v*/*v*) glutaraldehyde for ultrastructural observation. Some samples were immediately put into liquid nitrogen and then stored at −80 °C for gene expression detection.

### 2.3. The Detection of Oxidative Stress Indexes

Homogenized samples were used to measure H_2_O_2_ content and CAT, SOD, GPx, and GST activities with assay kits H_2_O_2_ (number: A064-1-1), CAT (number: A007-1-1), SOD (number: A001-1), GPx (number: A005-1-2), and GST (number: A004-1-1), respectively. The above kits were bought from Nanjing Jiancheng Bioengineering Institute (Nanjing, China).

### 2.4. Microstructural Observation

The samples fixed in 4% (*v*/*v*) paraformaldehyde were taken out and dehydrated with different concentrations (70%, 80%, 90%, 95%, and 100%) of ethanol. The dehydrated samples were embedded in paraffin to make wax blocks. The wax blocks were cut into 5–10 µm slices using a slicer manufactured by Jinhua Wireless Power Plant (Zhejiang, China). The obtained slices were dewaxed, stained with hematoxylin and eosin, and sealed with neutral gum. The obtained slides were scanned using a digital slice scanning system (WINMEDIC, Shandong, China) and observed using ZYFViewer (Shandong Zhiying Medical WINMEDIC Technology Co., Ltd., Jinan, China).

### 2.5. Ultrastructural Observation

The samples fixed in 2.5% (*v*/*v*) glutaraldehyde were taken out, rinsed with PBS (pH = 7.2, three times) for 15 min each time, and fixed with 1% (*v*/*v*) osmium for 2.5 h. Then, the samples were immersed in different concentrations of ethanol at 4 °C, immersed in 100% acetone at room temperature, and put in embedding holes. The embedding solution was slowly added to the embedding holes. Then, the embedded tissue blocks were placed in an oven (at 37 °C) overnight and sliced using a ultramicrotome (LR604, RMC company, San Diego, CA, USA). The obtained slides were stained with uranium acetate and lead citrate. The ultrastructures were observed under a transmission electron microscopy (GEM-1200ES, Japan Electron Optics Laboratory, Tokyo, Japan).

### 2.6. The Detection of Genes at the Transcriptional Level

#### 2.6.1. Primers

In order to further investigate the molecular mechanism of Se alleviating Pb poisoning, we measured the mRNA expressions of three Txnrds, four HSPs, and five apoptosis-related genes. In our experiment, the primer sequences were obtained from Genebank (www.ncbi.nlm.nih.gov/genbank/ (accessed on 6 January 2023)) and specific information is shown in Table S1. All primers were synthesized by Shanghai Yingjun Bioengineering Co., Ltd. (Shanghai, China). β-actin was selected as an internal reference gene.

#### 2.6.2. Total RNA Extraction and cDNA Synthesis

The samples frozen in a −80 °C fridge were taken out and ground in a mortar containing liquid nitrogen. TRIzol (Takara, Japan) was used to extract total RNA. The specific operation steps were the same as our previous research [21]. The obtained total RNA was dried at room temperature for the synthesis of complementary DNA (cDNA). Diethyl pyrocarbonate water was used to dissolve the total RNA. The dissolved total RNA was used to measure the OD260/OD280 ratio, and the obtained OD260/OD280 ratio (1.9–2.1) met the requirement of RNA purity. A cDNA synthesis system (20 μL) was prepared and put in a thermal cycler (T960, Hangzhou Jingke Instrument Co., Ltd., Zhejiang, China), set to operate at 30 °C for 10 min, at 42 °C for 1 h, and at 85 °C for 5 min for the reverse transcription of the cDNA. The obtained product was the cDNA which was used for PCR amplification. All reagents were obtained from Tiangen Biotech Co., Ltd. (Beijing, China).

#### 2.6.3. PCR Amplification

A PCR amplification system (20 μL) was prepared and put in a Realtime PCR instrument (AppLied Biosystems, Foster, Fullerton, CA, USA) for PCR amplification. The reaction condition was at 95 °C for 30 s (pre-denaturation), at 95 °C for 5 s (denaturation), and at 60 °C for 34 s (annealing and elongation). Five replicates were performed for each sample. The 7500 ReaL Time PCR System software v2.0 (AppLied Biosystems, Foster, Fullerton, CA, USA) was used to obtain raw data. The raw data were calculated using the 2^−ΔΔCt^ method [22,23] to obtain the relative mRNA expression.

### 2.7. The Calculation of Integrated Biomarker Response Value

To evaluate different effects of Pb on chicken cerebellums and thalami, the Integrated Biomarker Response (IBR) value was calculated. The calculation method was the same as [24]. The main steps included the standardization of the raw data, the calculation of each biomarker score, the visualization of the response level for each biomarker using a star chart, and finally, the calculation of the IBR value.

### 2.8. Multivariate Correlation Analysis and Principal Component Analysis 

We further analyzed the correlation between all factors using the multivariate correlation analysis method. The correlation coefficient diagrams were plotted using an online data analysis platform (http://www.bioinformatics.com.cn (accessed on 15 April 2023)). Principal Component Analysis (PCA) was performed to further analyze the relationship between the seventeen factors detected using the SPSS software v25.0 (Chicago, IL, USA).

### 2.9. Statistical Analyses

Our data passed the normality test and the homogeneity test of variance. The level of each factor is shown as the mean ± the standard deviation (*n* = 5) and were visualized using prism v6.0 (Graph-Pad Software v6.0, San Diego, CA, USA). Then, one-way ANOVA was used to analyze the significant differences of the data between different groups. Different lowercase letters and capital letters mean significant (*p* < 0.05) differences among different groups at the same time point and among different time points in the same group, respectively.

## 3. Results

### 3.1. Morphological Structural Changes

**Figure 1:**
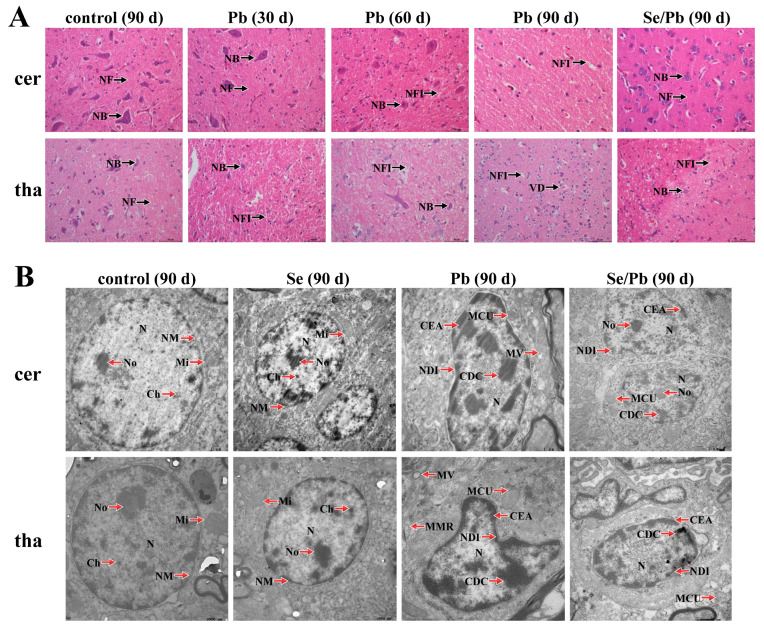
Microstructures (×400) and ultrastructures of chicken cerebellums and thalami. (**A**) microstructure; (**B**) ultrastructure. cer: cerebellums; tha: thalami. Control (90 d): the control group on Day 90; Pb (30 d): the Pb group on Day 30; Pb (60 d): the Pb group on Day 60; Pb (90 d): the Pb group on Day 90; Se/Pb (90 d): the Se/Pb group on Day 90; Se (90 d): the Se group on Day 90. NFs: Nerve Fibers, NB: Nissl Body, NFI: space among Nerve Fibers Increased, VD: Vacuolar Degeneration, N: Nucleus, NM: Nuclear Membrane, No: Nucleolus, Ch: Chromatin, Mi: Mitochondria, NDI: Nuclei Deformed and Irregular, CDC: Chromatin Deeply Colored, CEA: Chromatin Edge Aggregation, MCU: Mitochondrial Cristae Unclear, MMR: Mitochondrial Membrane Rupture, MV: Mitochondrial Vacuolization.

In order to investigate whether Pb can damage chicken cerebellums and thalami and whether Se can alleviate Pb-caused damage, microstructural (Figure 1A) and ultrastructural (Figure 1B) observations were performed. Regarding the microstructures of chicken cerebellums, in the control group on Day 90, the following phenomena were observed: tightly packed Nerve Fibers (NFs), as well as clear and dense Nissl Bodies (NBs). In the Pb group on Day 30, we observed that the Nissl bodies became smaller and fewer in number compared to the control group. In the Pb group on Day 60, the Nissl bodies had further shrunk and decreased and the space among the Nerve Fibers Increased (NFI) compared with the Pb group on Day 30. On Day 90 of Pb exposure, the Nissl bodies disappeared and the nerve fiber spaces were further enlarged. In the Se/Pb group on Day 90, the Nissl bodies were smaller than those in the control group on Day 90 and were more numerous than the Pb-exposure group on Day 90. The tightness of the nerve fibers was similar to that in the control group and increased compared with the Pb-exposure group on Day 90. Regarding the microstructure of thalami, in the control group on Day 90, the Nissl bodies were clear and the neurons were intact. In the Pb group on Days 30 and 60, the Nissl bodies shrunk. The nerve fiber space increased compared with the control group and increased with time. On Day 90, the Nissl bodies disappeared and the nerve cells showed Vacuolar Degeneration (VD). In the Se/Pb group on Day 90, the Nissl bodies could be observed; however, the size and number of the Nissl bodies was smaller and fewer, respectively, than those in the control group. The size of the nerve fiber space was between the control group and the Pb-treatment group on Day 90.

Regarding the ultrastructures of cerebellums and thalami (Figure 1B), on Day 90, in the control and Se groups, there were round Nuclei (Ns), clearly visible Nucleolus (No), fine and uniform Chromatin (Ch) particles, neat Nuclear Membranes (NM), and clear Mitochondrial (Mi) cristae in cerebellar and thalamic cells. In the Pb group, the Nuclei were Deformed and Irregular (NDI), nucleoli disappeared, Chromatin was Deeply Colored (CDC), Edge Aggregation (CEA) was serious, Mitochondrial Cristae were Unclear (MCU), and Mitochondrial Vacuolization (MV) was observed in both chicken cerebellums and thalami. In the Pb group on Day 90, the degree of nuclear deformation in thalami was more serious than that in cerebellums, and Mitochondrial Membrane Rupture (MMR) appeared in thalami. In the Se/Pb group on Day 90, nucleoli and unclear mitochondrial cristae can be seen and the degrees of nuclear deformation, chromatin aggregation, and marginal aggregation were lower than those in the Pb group on Day 90 in chicken cerebellums and thalami.

### 3.2. H_2_O_2_ Content and Antioxidant Enzyme Activities

**Figure 2:**
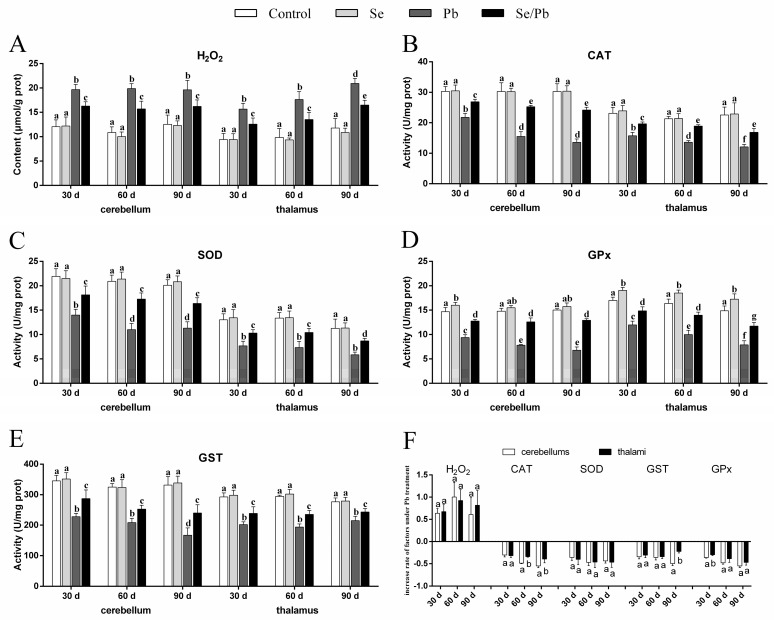
H_2_O_2_ content, four antioxidant enzymes’ (CAT, SOD, GST, and GPx) activities, and increase rate of the five factors in chicken cerebellums and thalami. (**A**) H_2_O_2_; (**B**) CAT; (**C**) SOD; (**D**) GST; (**E**) GPx; (**F**) increase rate of the five factors. Different lowercase letters at the top of the bars represent significant (*p* < 0.05) differences between different time points in the same group, between different groups at the same time point (**A**–**E**), and between cerebellums and thalami of the same factor at the same time points (**F**). Each value is shown as the mean ± the standard deviation (*n* = 5).

The content of H_2_O_2_ (Figure 2A), as well as the activities of four antioxidant enzymes (CAT (Figure 2B), SOD (Figure 2C), GST (Figure 2D), and GPx (Figure 2E)) were measured in cerebellums and thalami with kits. In our results, compared with the control group at all three time points, there were no significant (*p* > 0.05) changes of all five factors in the Se group, except that GPx activity increased significantly (*p* < 0.05) in cerebellums on Day 30 and in thalami on Days 30, 60, and 90. A significant (*p* < 0.05) increase of H_2_O_2_ content occurred in the Pb and Se/Pb groups compared with that in the control and Se groups at all three time points. Nevertheless, a significant (*p* < 0.05) decrease in the activities of the four antioxidant enzymes occurred in the Pb and Se/Pb groups compared with those in the control and Se groups. A significant (*p* < 0.05) increase in the activities of the four antioxidant enzymes occurred in the Se/Pb group compared with those in the Pb group. In addition, regarding the Pb group, on Day 90 compared with on Days 30 and 60, we found significantly (*p* < 0.05) up-regulated content of H_2_O_2_ in thalami; in contrast, there was a significant (*p* < 0.05) down-regulated activity of GST in cerebellums. On Days 60 and 90 compared with on Day 30, we found significant (*p* < 0.05) down-regulated activities of CAT, SOD, and GPx in cerebellums. With the increase of Pb-treatment time, we found a significant (*p* < 0.05) down-regulated trend in the activities of CAT and GPx in thalami. We further compared the change rates of all the detected factors in the Pb group with respect to the control group between cerebellums and thalami to explore the difference of Pb poisoning in cerebellums and thalami (Figure 2F). A significantly (*p* < 0.05) greater decrease rate was found for CAT on Days 60 and 90 and for GPx on Day 30 in cerebellums compared with those in thalami. A greater decrease rate (*p* > 0.05) was found for GST on Day 90 and for GPx on Days 60 and 90 in cerebellums compared with those in thalami.

### 3.3. mRNA Levels of Txnrds and HSPs

**Figure 3:**
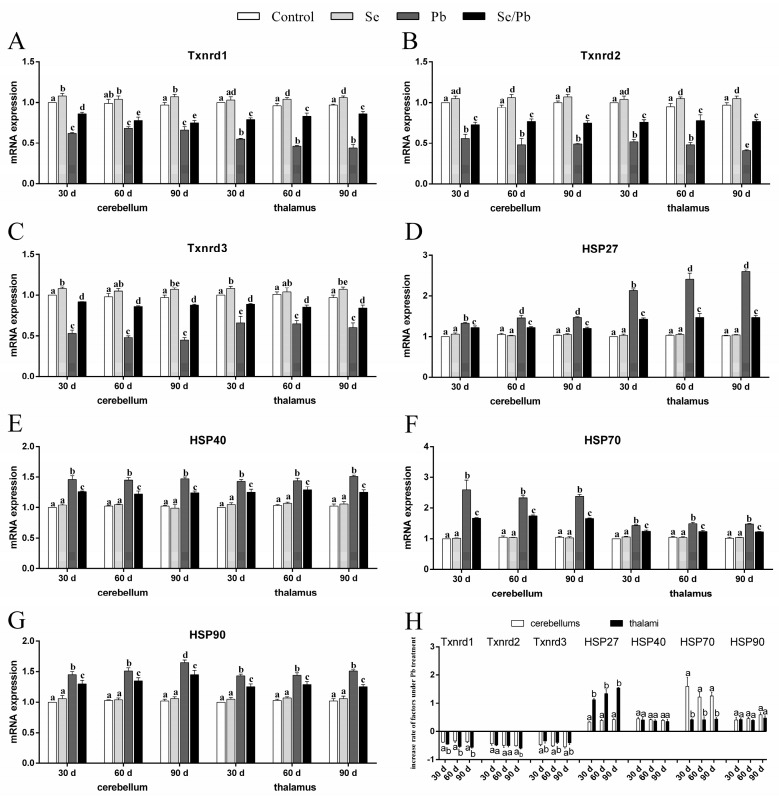
mRNA levels of the three Txnrds and four HSPs and the increase rate of the seven genes in chicken cerebellums and thalami. (**A**) Txnrd1; (**B**) Txnrd2; (**C**) Txnrd3; (**D**) HSP27; (**E**) HSP40; (**F**) HSP70; (**G**) HSP90; (**H**) increase rate of the seven genes. Different lowercase letters at the top of the bars represent significant (*p* < 0.05) differences between different time points in the same group, between different groups at the same time point (**A**–**G**), and between cerebellums and thalami of the same factor at the same time points (**H**). Each value is shown as the mean ± the standard deviation (*n* = 5).

qRT-PCR was used to explore three Txnrds (Txnrd1, Txnrd2, and Txnrd3) and four HSPs (HSP27, HSP40, HSP70, and HSP90) at the transcription level. As shown in Figure 3A–G, there were no significant (*p* > 0.05) differences in all seven genes detected between the control group and the Se group in both cerebellums and thalami at the three time points. However, the Se group showed significantly (*p* < 0.05) increased Txnrd1 in cerebellums on Days 30 and 90, in thalami on Days 60 and 90, increased Txnrd2 in both cerebellums and thalami on Days 60 and 90, as well as increased Txnrd3 in both cerebellums and thalami on Days 30 and 90 compared with the control group. The Pb group showed significantly (*p* < 0.05) reduced mRNA levels of the three detected Txnrds and significantly (*p* < 0.05) elevated levels of the four detected HSPs compared with the control, Se, and Se/Pb groups in cerebellums and thalami. The Se/Pb group showed a significant (*p* < 0.05) reduction of Txnrds and a significant (*p* < 0.05) elevation of HSPs compared with the control and Se groups in cerebellums and thalami. Additionally, regarding the Pb group, Txnrd2 mRNA expression on Day 90 was significantly (*p* < 0.05) lower than that on Days 30 and 60 in thalami. HSP27 mRNA expression on Days 60 and 90 was significantly (*p* < 0.05) higher than that on Day 30 both in cerebellums and thalami. HSP90 mRNA expression on Day 90 was significantly (*p* < 0.05) higher than that on Days 30 and 60 in cerebellums. Regarding the change rates of Txnrds and HSPs (Figure 3H), decreased rates of Txnrd1 at the three time points and Txnrd2 on Day 30 in thalami were significantly (*p* < 0.05) greater than those in cerebellums. The decrease rate of Txnrd2 in thalami was greater (*p* > 0.05) than that in cerebellums on Days 30 and 60. However, the decrease rate of Txnrd3 in cerebellums was significantly (*p* < 0.05) greater than that in thalami on Days 60 and 90. The increase rate of HSP27 in cerebellums was significantly (*p* < 0.05) greater than that in thalami; on the contrary, the increase rate of HSP70 in thalami was significantly (*p* < 0.05) greater than that in cerebellums at the three time points. There was no significant (*p* > 0.05) difference in the increase rates of HSP40 and HSP90 between cerebellums and thalami at the three time points.

### 3.4. mRNA Levels of Apoptosis-Related Genes

**Figure 4:**
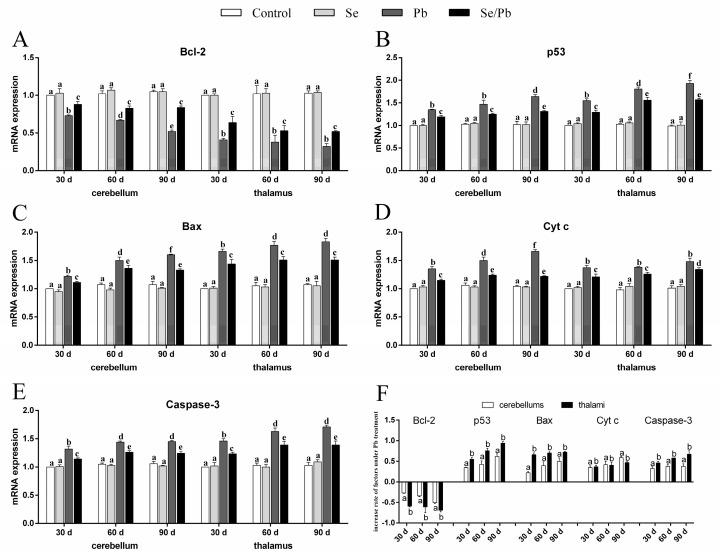
mRNA levels of five apoptosis-related genes and the increase rate of the five genes in chicken cerebellums and thalami. (**A**) Bcl-2; (**B**) p53; (**C**) Bax; (**D**) Cyt c; (**E**) Caspase-3; (**F**) increase rate of the five genes. Different lowercase letters at the top of the bar represent significant (*p* < 0.05) differences between different time points in the same group, between different groups at the same time point (**A**–**E**), and between cerebellums and thalami of the same factor at the same time points (**F**). Each value is shown as the mean ± the standard deviation (*n* = 5).

We detected the mRNA levels of five apoptosis-related genes (Bcl-2, p53, Bax, Cyt c, and Caspase-3), in order to investigate the molecular mechanism of Pb-induced apoptosis and Se-alleviated apoptosis caused by Pb. The obtained results were as follows (Figure 4A–E). At all the three time points, all five detected apoptosis-related genes were not significantly different (*p* > 0.05) between the control group and the Se group in cerebellums and thalami. Bcl-2 in the Pb and Se/Pb groups was significantly (*p* < 0.05) lower than that in the control and Se groups. Bcl-2 in the Se/Pb group was significantly (*p* < 0.05) higher than that in the Pb group. On the contrary, the other four apoptosis-related genes in the Pb and Se/Pb groups were significantly (*p* < 0.05) higher than those in the control and Se groups. The four apoptosis-related genes in the Se/Pb group were significantly (*p* < 0.05) lower than those in the Pb group. Regarding the Pb group at the three time points, with the extension of Pb-exposure time, Bcl-2 in cerebellums was down-regulated significantly (*p* < 0.05) and in thalami was down-regulated (*p* > 0.05). p53 in cerebellums and thalami was up-regulated significantly (*p* < 0.05), except that an insignificant (*p* > 0.05) up-regulation was found in cerebellums on Day 60 compared with that on Day 30. Bax in cerebellums and thalami was up-regulated significantly (*p* < 0.05), except that an insignificant (*p* > 0.05) up-regulation was found in thalami on Day 90 compared with that on Day 60. Cyt c in cerebellums was up-regulated significantly (*p* < 0.05) and in thalami was up-regulated (*p* > 0.05). Caspase-3 in cerebellums and thalami was up-regulated significantly (*p* < 0.05), except that an insignificant (*p* > 0.05) up-regulation was found both in cerebellums and thalami on Day 90 compared with that on Day 60. Regarding change rates (Figure 4F), the decrease rate of Bcl-2, and increase rates of p53, Bax, and Caspase-3 in thalami were significantly (*p* < 0.05) greater than those in cerebellums at the three time points. There was no significant (*p* > 0.05) difference in the increase rate of Cyt c between cerebellums and thalami on Days 30 and 60. The increase rate of Cyt c in cerebellums was significantly (*p* < 0.05) greater than that in thalami on Day 90.

### 3.5. IBR Values for Txnrds, Oxidative Stress Indexes, HSPs, and Apoptosis-Related Genes

**Figure 5:**
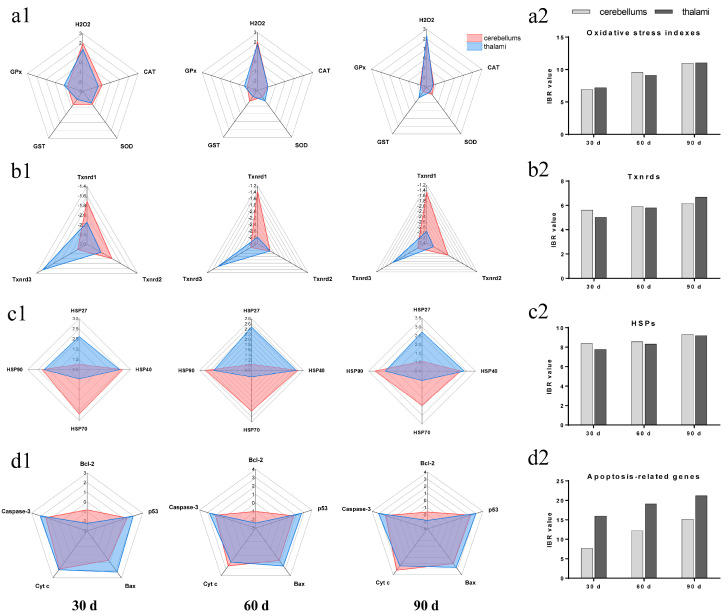
Response level of all the detected factors and IBR value of oxidative stress indexes, Txnrds, HSPs, and apoptosis-related genes in chicken cerebellums and thalami under Pb exposure. (**a1**) Response level of oxidative stress indexes; (**a2**) IBR value of oxidative stress indexes; (**b1**) response level of Txnrds; (**b2**) IBR value of Txnrds; (**c1**) response level of HSPs; (**c2**) IBR value of HSPs; (**d1**) response level of apoptosis-related genes; (**d2**) IBR value of apoptosis-related genes.

In our experiment, we found that Pb exposure led to toxic damage to cerebellums and thalami in chickens. Hereby, the IBR value was used to compare the Pb toxicity degree between cerebellums and thalami from the perspective of four molecular mechanisms (Txnrds, oxidative stress, HSPs, and apoptosis). The response levels and IBR values for the factors measured in our research were calculated at the three time points (Figure 5). Regarding the oxidative stress indexes (Figure 5(a1,a2)), the response levels for H_2_O_2_ on Day 90, CAT at the three time points, SOD on Days 30 and 90, GST on Days 30 and 60, and GPx on Day 90 in thalami were higher than those in cerebellums. On the contrary, the response levels for H_2_O_2_ on Days 30 and 60, SOD on Day 60, GST on Day 90, and GPx on Days 30 and 60 in thalami were lower than those in cerebellums (Figure 5(a1)). The IBR values for the oxidative stress indexes in cerebellums on Days 30 and 90 were higher than those in thalami. The IBR values increased with time in both cerebellums and thalami (Figure 5(a2)). Regarding Txnrds (Figure 5(b1,b2)), the response levels for Txnrd1 at the three time points and Txnrd2 on Days 30 and 90 in thalami were higher than those in cerebellums; on the contrary, the response levels for Txnrd3 at the three time points in thalami were lower than those in cerebellums (Figure 5(b1)). The IBR values for all three Txnrds in cerebellums on Day 30 and in thalami on Day 90 were higher than those in thalami and cerebellums, respectively. The IBR values for all three Txnrds showed an increase trend in both cerebellums and thalami (Figure 5(b2)). Regarding HSPs (Figure 5(c1,c2)), the response levels for HSP27 at the three time points and HSP40 on Day 90 in thalami were higher than those in cerebellums. On the contrary, the response levels for HSP40 on Days 30 and 60 and HSP70 and HSP90 at the three time points in thalami were higher than those in cerebellums (Figure 5(c1)). The IBR values for HSPs in cerebellums at the three time points were higher than those in thalami. The IBR values for HSPs increased with time in both cerebellums and thalami (Figure 5(c2)). Regarding apoptosis-related genes (Figure 5(d1,d2)), all five apoptosis-related genes’ response levels in thalami were higher than those in cerebellums at all three time points, except that Cyt c’s response level in thalami was lower than that in cerebellums on Days 60 and 90 (Figure 5(d1)). The IBR values for apoptosis-related genes in thalami at the three time points were higher than those in cerebellums and showed an increasing trend with the increase of the Pb-treatment time in both cerebellums and thalami (Figure 5(d2)).

### 3.6. Correlation Matrix of All Measured Factors

**Figure 6:**
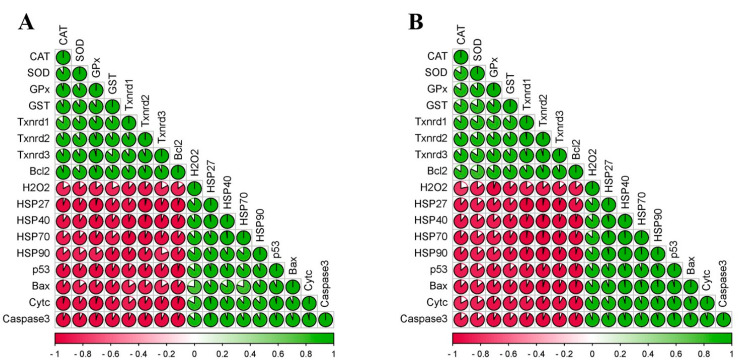
Correlation matrix of all seventeen factors detected in chicken cerebellums and thalami. (**A**) Cerebellum; (**B**) thalamus. Different colors represent relational values.

In order to investigate the relationship among all seventeen factors measured, the multiple correlation analysis method was performed and correlation coefficients among the seventeen factors were calculated in chicken cerebellums and thalami. The obtained correlation coefficient diagrams of chicken cerebellums (Figure 6A) and thalami (Figure 6B) showed that there was a positive correlation at the 0.01 level among 8 factors (CAT, SOD, GPx, GST, three Txnrds, and Bcl-2), as well as among 9 factors (H_2_O_2_, four HSPs, p53, Bax, Cyt c, and Caspase-3). Furthermore, we also found that there was a negative correlation at the 0.01 level between the 8 factors and the 9 factors mentioned above.

### 3.7. PCA Results of the Seventeen Factors

The PCA method was used in order to further explore the possible correlation of all seventeen factors detected in cerebellums and thalami. As shown in Table 1, the PCA results indicated that the total initial eigenvalues for Component 1 were 15.599 and 15.686 in cerebellums and thalami, respectively, which were higher than 1 in cerebellums and thalami. Nevertheless, the initial eigenvalues of the other sixteen components were less than 1. Our above findings mean that only Component 1 met the standard for extracting principal components. Furthermore, the variance of Component 1 was 91.758% and 92.269% in chicken cerebellums and thalami, respectively, as well as was more than 80%, which meant that Component 1 included enough data information for both cerebellums and thalami, respectively. As shown in Table S2, the obtained Component 1 explained over 90% of information in each factor, which meant that all seventeen factors belonged to Component 1 in both cerebellums and thalami. Our above findings indicated that only one component can be extracted both for cerebellums and thalami, so the relationship of the seventeen factors detected was close.

## 4. Discussion

Excessive Pb can impair chicken health. Two reports mentioned that Pb exposure damaged chicken muscles [25] and cerebrums [8] via morphological observation. Microstructural and ultrastructural observations are morphological observation methods for studying the damage to organisms caused by poisoning [26,27,28]. In our experiment, the microstructural observation indicated that excessive Pb caused cerebellar and thalamic injury in chickens, such as smaller and fewer Nissl bodies. Moreover, we found that the injury was time-dependent. Li et al. (2021) also found that excessive cobalt reduced Nissl bodies in mice [29]. In addition, our ultrastructural observation indicated that chickens who received Pb showed apoptotic damage, such as nuclear membrane contraction, nuclear deformation, the aggregation and marginalization of chromatin, unclear mitochondrial cristae, and mitochondrial vacuolization in cerebellums and thalami. The previous four research works reported similar phenomena. Cadmium-exposed common carp kidneys exhibited nuclear deformation, chromatin marginalization, and apoptotic damage [30]. Pb treatment resulted in chromatin aggregation and apoptotic injury in Japanese quail thymus [31]. Excessive arsenic led to apoptotic damage with aggregative chromatin and unclear mitochondrial crest in chicken thalami [32]. The cerebrums of chickens exposed to Pb showed apoptotic damage with nuclear deformation and chromatin marginalization [8]. Interestingly, in our experiment, after the chickens were treated with Pb, thalami showed vacuolar degeneration and mitochondrial membrane rupture. The degrees of nuclear deformation and chromatin aggregation in thalami were more serious than those in cerebellums. Our discovery by microstructural and ultrastructural observation means that, under Pb treatment, thalamic apoptotic injury was more serious than cerebellar apoptotic injury. This was the first study to compare the degree of damage in the case of poisoning between two parts of the brain, which needs to be further explored. In addition, studies have found that Se can relieve the neurotoxicity and apoptotic damage caused by hazardous substances. Gholamigeravand et al. (2021) reported that Se mitigated cerebral toxicity induced by streptozotocin in rats [33]. Se supplementation ameliorated cerebral injury caused by mercury and Pb in chickens [8,34]. An investigation recorded that Se alleviated apoptotic damage caused by the environmental pollutant acrolein in rat livers [35]. Similar to the above findings, we also found that Se supplementation relived Pb-induced apoptotic damage in both chicken cerebellums and thalami.

Recent studies found that oxidative stress was involved in the molecular mechanism of Pb-caused cerebellar injury in mice [36] and thymus injury in quails [31]. H_2_O_2_, CAT, SOD, GST, and GPx are oxidative stress indexes. H_2_O_2_ is a kind of reactive oxygen that can promote oxidation. CAT, SOD, GPx, and GST belong to the antioxidant enzyme system. The four factors can decompose H_2_O_2_, remove superoxide, reduce peroxide into non-toxic hydroxyl compounds, and remove peroxide, respectively. In our experiment, Pb treatment decreased CAT, SOD, GST, and GPx activities at the three time points; on the contrary, H_2_O_2_ content increased, which indicated that excessive Pb reduced the antioxidant capacity, increased the reactive oxygen, and caused oxidation/antioxidation imbalance and oxidative stress in chicken cerebellums and thalami. Other reports also revealed that exposure to heavy metals down-regulated CAT, SOD, GST, and GPx activities, up-regulated H_2_O_2_ content, and caused oxidation/antioxidation imbalance and oxidative stress in animals [21,37,38]. Moreover, our findings showed a down-regulated time dependency of CAT and GPx in chicken thalami. In addition, we found a greater inhibition degree of CAT in thalami compared to cerebellums under Pb exposure, which needs to be further explored. Interestingly, Pb can bind to the sulfhydryl group of proteins, and the four antioxidant enzymes we tested (CAT, SOD, GST, and GPx) contain a sulfhydryl group [39]. Thus, in our experiment, Pb might bind to the sulfhydryl group of CAT, SOD, GST, and GPx, resulting in the inactivation of the above four antioxidant enzymes, which further supports that excessive Pb reduced antioxidant capacity through binding CAT, SOD, GST, and GPx in chicken cerebellums and thalami, which needs further investigation.

Txnrds (including Txnrd1, Txnrd2, and Txnrd3), a class of selenoproteins, are three thioredoxin reductases. Three researchers demonstrated that the knockdown of Txnrd1 resulted in decreased Txnrd1 and oxidative stress in human CML cells [40]; the knockdown of Txnrd2 decreased Txnrd2, CAT, SOD, and GPx, as well as caused oxidative stress in NSCLC cells [41]; the knockout of Txnrd3 aggravated nickel-induced cardiac oxidative stress in mice [42]. Hence, we further measured Txnrd1, Txnrd2, and Txnrd3. At the three time points, Pb treatment decreased the above three Txnrds’ mRNA expression. Thus, our above findings further demonstrated that Pb exposure reduced antioxidant capacity, caused oxidative stress via decreasing Txnrds, and Txnrds mediated Pb-caused oxidative stress in chicken cerebellums and thalami. For the first time, we found that Txnrds mediated the oxidative stress induced by heavy metals, which needs to be investigated in the future. It is important to note that both the decrease rate and response level of Txnrd1 in thalami were greater than those in cerebellums, and the IBR value indicated that, on Day 90, the inhibition of Txnrds in thalami was greater than that in cerebellums, further supporting our morphological results: Pb-caused thalamic injury was more serious than cerebellar injury in chickens.

Nutrients have antioxidant capacity [43], such as the nutrient element Se. A study conducted by Sun et al. (2020) indicated that Se pre-treatment increased GPx activity in an oxidative stress model of rat cardiomyocytes [44]. Cd-induced oxidative stress with increased H_2_O_2_ and decreased SOD and GPx was alleviated by Se supplementation in laying hen ovaries [45]. Se supplementation increased CAT and GST, decreased H_2_O_2_, and reduced oxidative stress in the testes of chickens during Pb treatment [14]. We found that CAT, SOD, GST, GPx, Txnrd1, Txnrd2, and Txnrd3 were higher and H_2_O_2_ was lower in the Se/Pb group compared with those in the Pb group at the three time points both in chicken cerebellums and thalami. Our results mean that Se supplementation ameliorated Pb-caused oxidative stress through up-regulating Txnrds, which needs to be explored more. Furthermore, our multivariate correlation analysis results demonstrated that there was a positive relationship (at the 0.01 level) between Txnrd1, Txnrd2, Txnrd3, CAT, SOD, GST, and GPx, which also supported our findings. In addition, Bi et al. (2019) demonstrated that Se and Pb formed a complex in mouse liver cells [46]. Se supplementation decreased the accumulation of Pb in the blood, bone, and livers and increased the excretion of Pb in urine and feces in rats [47]. GPx, Txnrd1, Txnrd2, and Txnrd3 contain Se and showed higher levels in the Se group and the Se/Pb group than those in the control group and the Pb group, respectively. Thus, in our experiment, the mechanism by which Se alleviates Pb-induced neurotoxicity may be: GPx, Txnrd1, Txnrd2, and Txnrd3 bound to Pb to form a Pb-Se complex, which is excreted from the body and reduces Pb toxicity, which needs further study.

Dong et al. (2021) indicated that excessive Cd induced both oxidative stress and apoptosis in HTR-8/SVneo cells [48]. We also explored apoptosis. Multiple genes are involved in the molecular mechanism of apoptosis, such as p53, Bcl-2, Bax, Cyt c, and Caspase-3. p53 inhibitor pifithrin-α inhibited ropivacaine and caused the decrease of Bcl-2 (an anti-apoptotic gene) and the increase of p53 and Bax (a pro-apoptotic gene) in PC12 cells [49]. Bcl-2 and Bax are located on the outer membrane of mitochondria and can decrease and increase membrane permeability, respectively, which prevents and promotes the release of pro-apoptotic factor Cyt c, respectively. The released Cyt c can activate Caspase-3 and induce apoptosis. Hence, we measured p53, Bcl-2, Bax, Cyt c, and Caspase-3 and found that Pb up-regulated p53, Bax, Cyt c, and Caspase-3, down-regulated Bcl-2, and induced apoptosis in chicken cerebellums and thalami at the three time points. This also means that mitochondria participated in Pb-caused apoptosis via the p53-Cyt c-Caspase-3 pathway. Other research has supported our findings. Elevated p53 and an increased apoptosis level were found in arsenic-poisoned rat livers [50]. Cd increased Bax and Caspase-3, decreased Bcl-2, and led to apoptosis in pig livers [51]. Furthermore, we also found a time-dependent decrease of Bcl-2 and a time-dependent increase of Bax and Cyt c in cerebellums, as well as a time-dependent increase of p53 in thalami in Pb-exposed chickens. Interestingly, our findings revealed that Pb-exposed chickens showed a greater decrease rate of Bcl-2 and a greater increase rate of p53, Bax, and Caspase-3 in thalami than those in cerebellums. Moreover, the IBR value for the five apoptosis-related genes in thalami was higher than that in cerebellums. Our above findings indicated that the thalamic apoptotic degree was more serious than the cerebellar apoptotic degree in chickens treated with Pb, which further supported our morphological and oxidative stress results. In addition, the CAT supplement inhibited oxidative stress, the elevation of p53, and apoptosis in JB6 Cl41 cells treated by Cd [52]. H_2_O_2_ decreased Bcl-2, increased Bax and Caspase-3, and caused oxidative stress and apoptosis in human thyroid cells and thyroid fibroblasts [53]. Herein, our findings suggested that oxidative stress mediated apoptosis in Pb-treated chicken cerebellums and thalami via triggering p53-Cyt c-Caspase-3.

Se can resist apoptosis caused by adverse factors. Se pre-treatment relieved tetrahydrocannabinol-increased p53 and tetrahydrocannabinol-induced apoptosis in Mus Musculus Sertoli cells [54]. Se antagonized Pb-induced apoptosis by up-regulating Bcl-2 and down-regulating Bax and Cyt c in both RAW264.7 and PC12 cells [55]. In our experiment, we also found an improvement of the abnormalities of apoptosis-related genes (p53, Bcl-2, Bax, Cyt c, and Caspase-3) caused by Pb at the three time points. It can be concluded that Se alleviated Pb-induced apoptosis via alleviating oxidative stress. The p53-Cyt c-Caspase-3 pathway took part in alleviating the mechanism of Se on oxidative stress-mediated apoptosis in Pb-exposed chicken cerebellums and thalami.

Arsenic caused oxidative stress and apoptosis with inhibited CAT and Bcl-2, as well as elevated HSP70, HSP90, p53, Bax, and Caspase-3 in chicken brains [56]. Excessive manganese exposure increased HSP27, HSP40, HSP70, and HSP90, and injured chicken cerebrum [18]. Thus, we further explored HSP27, HSP40, HSP70, and HSP90 and found that Pb treatment elevated mRNA levels of the four HSPs at the three time points in chicken cerebellums and thalami, meaning that HSPs partook in Pb-caused damage in chicken cerebellums and thalami. Interestingly, in the Pb group, our results showed that the increase rate and response level for HSP27 in thalami were higher than those in cerebellums; on the contrary, the increase rate and response level for HSP70 in thalami were lower than those in cerebellums. Our above findings suggested that, in chickens, HSP27 in thalami was more sensitive than that in cerebellums and HSP70 in cerebellums was more sensitive than that in thalami under Pb exposure, which needs deeper exploration. In addition, oxidative stress inducer 15d-PGJ2 caused oxidative stress and up-regulated HSP70 in rat chondrocytes, and antioxidant N-acetylcysteine supplementation inhibited 15d-PGJ2-increased HSP70 in rat chondrocytes [57]. The supplementation of H_2_O_2_ led to oxidative stress and up-regulated HSP90 content in HUVEC cells [58]. Antioxidant 7,8-dihydroxy-4-methylcoumarin treatment reversed H_2_O_2_-increased HSP90 in HUVEC cells [58]. Therefore, our findings suggested that Pb-induced oxidative stress stimulated HSP70 and HSP90. Interestingly, a study demonstrated that the knockdown of HSP70 decreased apoptosis in peripheral blood lymphocytes of piglets [59]. Accordingly, our results suggested that HSP70 mediated apoptosis induced by Pb poisoning in chicken cerebellums and thalami. Furthermore, HSP70 can be located on the mitochondrial membrane [60]. This means that HSP70 took part in the mechanism of mitochondria-involved Pb poisoning in chicken cerebellums and thalami.

Additionally, Se treatment reversed the four Pb-increased HSPs in our results, which means that HSPs were involved in the molecular mechanisms of Se relieving Pb-induced damage, also indicating that Se supplementation ameliorated Pb-induced oxidative stress and further decreased the Pb-increased HSP70 and HSP90, and finally, decreased HSP70 alleviated Pb-caused apoptosis in chicken cerebellums and thalami. Previous studies were consistent with our findings. Se supplementation reduced Pb-elevated HSP27, HSP40, HSP70, and HSP90 in chicken spermatogonia [7]. Se supplementation inhibited the Cd-induced decrease of CAT, SOD, GPx, and Bcl-2 and Cd-increased HSP27, HSP40, HSP70, HSP90, p53, Bax, Cyt c, and Caspase-3 in chicken hepatocytes [61]. In addition, the results of both the multivariate correlation and PCA analyses in our research also supported our above findings. The multivariate correlation analysis indicated that all factors had a 0.01 level of correlation. Only one principal component could be extracted using the PCA method. The results of the multivariate correlation and PCA proved that the seventeen factors were closely related, further confirming the complex molecular mechanism by which Se alleviated Pb-induced apoptosis in chicken cerebellums and thalami. Similar to our study, in the livers of excessive manganese-treated chickens, one principal component from the data of one oxidative stress index, five HSPs, and six inflammatory-related factors was extracted, which supported the molecular mechanism of excessive manganese induced hepatotoxicity: HSPs partook in oxidative stress-mediated inflammation [62].

## 5. Conclusions

**Figure 7:**
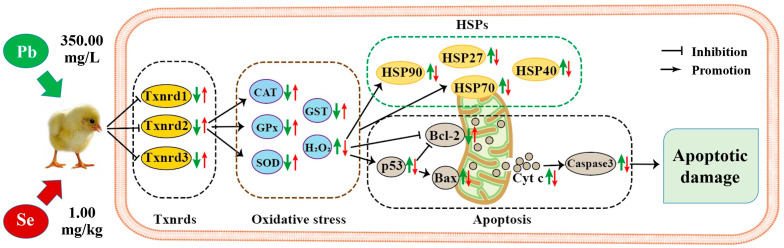
Model of Se relieving Pb toxicity in chicken cerebellums and thalami. “
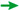
” represents the impact of Pb on the detected factors, and “
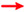
” represents the impact of Se supplementation on the detected factors.

To sum up, the model of our experiment on Se alleviating Pb toxicity in chicken cerebellums and thalami is shown in Figure 7. We found that Pb treatment resulted in apoptotic damage of the cerebellum and thalamus. Furthermore, excessive Pb decreased the anti-apoptotic gene Bcl-2 and increased four pro-apoptotic genes (p53, Bax, Cyt c, and Caspase-3) and four HSPs (HSP27, HSP40, HSP70, and HSP90), indicating that the p53-Cyt c-Caspase-3 pathway and HSPs took part in the mechanism of Pb-caused apoptotic injury. In addition, Pb increased H_2_O_2_, decreased four antioxidant enzymes (CAT, SOD, GPx, and GST), and three Txnrds (Txnrd1, Txnrd2, and Txnrd3), and caused oxidative stress via oxidation/antioxidation imbalance. Pb-inhibited Txnrds mediated oxidative stress, and Pb-induced oxidative stress increased HSPs. Furthermore, mitochondria participated in Pb-caused apoptosis via the p53-Cyt c-Caspase-3 pathway, and moreover, Pb-increased HSP70 triggered apoptosis. Interestingly, there was a time dependency for Bcl-2, Bax, and Cyt c in cerebellums, as well as for CAT, GPx, and p53 in thalami after chickens suffered Pb toxicity. The remarkable point is that we first found that Pb-caused apoptotic injury of thalami was more serious than that of cerebellums, which needs to be further explored. Interestingly, all the above Pb-induced changes of the seventeen factors were reversed by Se supplementation, meaning that Se supplementation alleviated Pb-induced apoptosis via the mitochondrial pathway in chicken cerebellums and thalami. Our findings add to the understanding of Pb-induced toxicity and provide new evidence for Se as an antidote.

## Figures and Tables

**Table 1:** PCA results of the seventeen factors in chicken cerebellums and thalami.

Organs	Principal Component	Initial Eigenvalues			Extraction Sums of Squared Loadings		
		Total	% of Variance	Cumulative %	Total	% of Variance	Cumulative %
cerebellum	1	14.644	91.523	91.523	14.644	91.523	91.523
	2	0.365	2.283	93.805			
	3	0.302	1.886	95.692			
	4	0.152	0.948	96.639			
	5	0.143	0.892	97.532			
	6	0.100	0.624	98.155			
	7	0.074	0.464	98.619			
	8	0.055	0.345	98.963			
	9	0.051	0.317	99.280			
	10	0.032	0.199	99.479			
	11	0.031	0.191	99.671			
	12	0.014	0.090	99.761			
	13	0.013	0.083	99.844			
	14	0.012	0.075	99.919			
	15	0.008	0.051	99.970			
	16	0.005	0.030	100.000			
thalamus	1	14.542	90.884	90.884	14.542	90.884	90.884
	2	0.409	2.558	93.442			
	3	0.312	1.950	95.392			
	4	0.160	0.997	96.390			
	5	0.149	0.932	97.322			
	6	0.117	0.729	98.051			
	7	0.093	0.583	98.634			
	8	0.066	0.415	99.049			
	9	0.047	0.296	99.344			
	10	0.033	0.204	99.549			
	11	0.025	0.158	99.707			
	12	0.019	0.118	99.825			
	13	0.017	0.104	99.929			
	14	0.006	0.040	99.968			
	15	0.004	0.026	99.995			
	16	0.001	0.005	100.000			

## Data Availability

Data sharing not applicable.

## Supplementary Materials

The following supporting information can be downloaded at: https://www.mdpi.com/article/10.3390/toxics11090734/s1, Table S1: Primer information of detected genes in chicken cerebellum and thalami; Table S2: Component matrix of seventeen factors in chicken cerebellums and thalami.

## Abbreviations

Lead (Pb); Selenium (Se); B-cell lymphoma-2 (Bcl-2); Bcl-2 associated X protein (Bax); Cadmium (Cd); Cytochrome c (Cyt c); Superoxide Dismutase (SOD); Catalase (CAT); Glutathione Peroxidase (GPx); Glutathione-S-Transferase (GST); Hydrogen Peroxide (H_2_O_2_); Thioredoxin Reductases (Txnrds); Heat Shock Protein (HSP); Multivariate correlation analysis and Principal Component Analysis (PCA).
