# Supplementary material for: Molecular Mechanisms of Selenium Mitigating Lead Toxicity in Chickens via Mitochondrial Pathway: Selenoproteins, Oxidative Stress, HSPs, and Apoptosis
Source: Toxics. 2023 Aug 25;11(9):734. doi: 10.3390/toxics11090734 (PMC10536545; doi:10.3390/toxics11090734)
Supplement: Supplementary file 1 [file toxics-11-00734-s001.zip › toxics-2479501-supplementary.pdf]

toxics MDPI

Supplementary Materials: Molecular Mechanisms of Selenium Mitigating Lead Toxicity in Chickens via Mitochondrial Pathway: Selenoproteins, Oxidative Stress, HSPs, and Apoptosis

Weichen Hong, Yuhao Liu, Jiatian Liang, Chunyu Jiang, Meijin Yu, Wei Sun, Bin Huang, Na Dong, Lu Kang and You Tang

Table S1. Primer information of detected genes in chicken cerebellum and thalami.

Genes	Serial numbers	Primer sequences	
**β-actin**	L08165	F: 5'-CCGCTCTATGAAGGCTACGC-3'	R: 5'-CTC TCG GCT GTG GTGGTG AA-3'
Txnrd1	NM_001030762.2	F: 5'-TACGCCTCTGGGAAATTCGT-3'	R: 5'-CTTGCAAGGCTTGTCCCAGTA-3'
Txnrd2	NM_001122691.1	F: 5'-GCTCTTAAAGATGCCCAGCACTAC-3'	R: 5'-GAACAGCTTGAGCCATCACAGA-3'
Txnrd3	NM_001122777.1	F: 5'-CCTGGCAAAACGCTAGTTGTG-3'	R: 5'-CGCACCATTACTGTGACATCTAGAC-3'
HSP27	NM_205290	F: 5′-ACACGAGGAGAAACAGGATGAG-3′	R: 5′- ACTGGATGGCTGGCTTGG-3′
HSP40	NM_001199325	F: 5′- GGGCATTCAACAGCATAGA-3′	R: 5′- TTCACATCCCCAAGTTTAGG-3′
HSP70	NM_001006685	F: 5'- CGGGCAAGTTTGACCTAA-3	R: 5'- TTGGCTCCCACCCTATCTCT-3′
HSP90	NM_001109785	F: 5'- TCCTGTCCTGGCTTTAGTTT-3′	R: 5′-AGGTGGCATCTCCTCGGT-3′
Bcl-2	NM_205339.1	F: 5'-ATCGTCGCCTTCTTCGAGTT-3'	R: 5'-ATCCCATCCTCCGTTGTCCT-3'
p53	NM_205264.1	F: 5'-GAGATGCTGAAGGAGATCAATGAG-3'	R: 5'-GTGGTCAGTCCGAGCCTTTT-3'
Bax	FJ977571.1	F: 5'-GATGAAGCCACCCAGCAGTA-3'	R: 5'-TGGATTCTCACAGTAGGAGGATG-3'
Cyt c	K02303.1	F: 5'-AGGCAAGCACAAGACTGGA-3'	R: 5'-CTGACTATCACCAAGAACCACC-3'
Caspase-3	NM_204725.1	F: 5'-CATCTGCATCCGTGCCTGA-3'	R: 5'-CTCTCGGCTGTGGTGGTGAA-3'

Table S2. Component matrix of seventeen factors in chicken cerebellums and thalami.

	Factors	H_2O_2	CAT	SOD	GPx	GST	Bcl-2	p53	Bax	Cyt c	Caspase-3	Txnrd1	Txnrd2	Txnrd3	HSP27	HSP70	HSP90
cerebellum	PC1	-0.902	0.96	0.944	0.974	0.949	0.975	-0.974	-0.93	-0.973	-0.984	0.949	0.97	0.951	-0.986	-0.947	-0.936
thalamus	PC1	0.911	-0.913	-0.866	-0.919	-0.943	-0.957	0.962	0.982	0.961	0.973	-0.969	-0.976	-0.962	0.981	0.986	0.983
